# Effects of mineralization on the hierarchical organization of collagen—a synchrotron X-ray scattering and polarized second harmonic generation study

**DOI:** 10.1098/rsfs.2023.0046

**Published:** 2024-06-07

**Authors:** Keke Zheng, Jingxiao Zhong, Jingrui Hu, Eve Nebbiolo, Juan Sanchez-Weatherby, Tengteng Tang, William J. Landis, Junning Chen, Peter Winlove, Benjamin E. Sherlock, James Bell

**Affiliations:** ^1^ Biomedical Engineering, Faculty of Environment, Science and Economy, University of Exeter, Exeter, UK; ^2^ Institute for Mechanical Process and Energy Engineering, School of Engineering and Physical Sciences, Heriot-Watt University, Edinburgh, UK; ^3^ School of Aerospace, Mechanical and Mechatronic Engineering, University of Sydney, Sydney, Australia; ^4^ VMX-I beamline, Diamond Light Source, Didcot, UK; ^5^ Materials Science & Engineering, McMaster University, Hamilton, Ontario, Canada; ^6^ Preventive and Restorative Dental Sciences, School of Dentistry, University of California at San Francisco, San Francisco, CA, USA; ^7^ Physics and Astronomy, Faculty of Environment, Science and Economy, University of Exeter, Exeter, UK; ^8^ School of Optometry and Vision Sciences, Cardiff University, Cardiff, UK

**Keywords:** collagen, mineralization, X-ray diffraction, second harmonic generation, nonlinear microscopy, polarization resolved

## Abstract

The process of mineralization fundamentally alters collagenous tissue biomechanics. While the structure and organization of mineral particles have been widely studied, the impact of mineralization on collagen matrix structure, particularly at the molecular scale, requires further investigation. In this study, synchrotron X-ray scattering (XRD) and polarization-resolved second harmonic generation microscopy (pSHG) were used to study normally mineralizing turkey leg tendon in tissue zones representing different stages of mineralization. XRD data demonstrated statistically significant differences in collagen D-period, intermolecular spacing, fibril and molecular dispersion and relative supramolecular twists between non-mineralizing, early mineralizing and late mineralizing zones. pSHG analysis of the same tendon zones showed the degree of collagen fibril organization was significantly greater in early and late mineralizing zones compared to non-mineralizing zones. The combination of XRD and pSHG data provide new insights into hierarchical collagen–mineral interactions, notably concerning possible cleavage of intra- or interfibrillar bonds, occlusion and reorganization of collagen by mineral with time. The complementary application of XRD and fast, label-free and non-destructive pSHG optical measurements presents a pathway for future investigations into the dynamics of molecular scale changes in collagen in the presence of increasing mineral deposition.

## 1. Introduction

**Figure 1.**
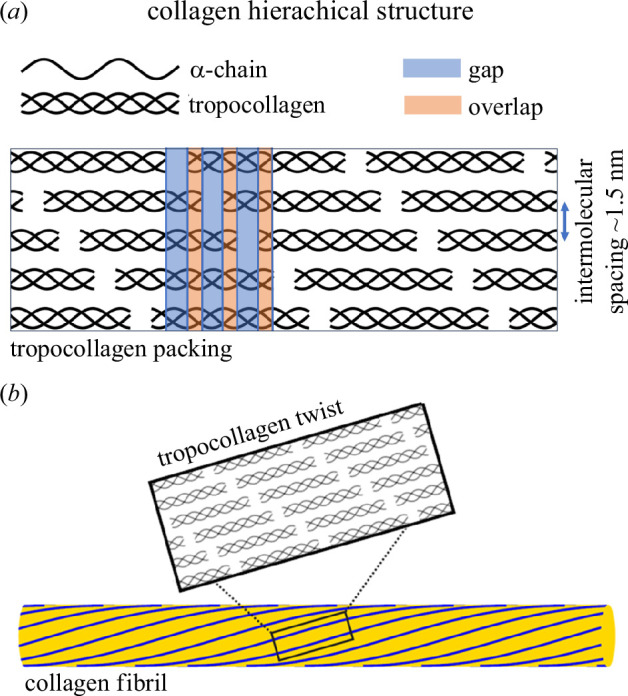
Diagram illustrating the hierarchical structure of type I collagen. (*a*) two-dimensional representation of the gap-overlap structure of a microfibril comprising five tropocollagen molecules in a staggered array. (*b*) illustration showing the supramolecular twist relative to the fibril axis.

Collagen is the most abundant structural protein in the mammalian body and plays a critical role in sustaining and transferring load in connective tissues to support a diverse range of mechanical functions *in vivo*. The structure of collagen is complex and hierarchical. At the molecular scale, type I collagen, the most common of the large and well-known collagen family of proteins, consists of three left-handed helical α-chains which self-assemble to form the right-handed triple helix, tropocollagen. Tropocollagen molecules are organized end-to-end in a staggered ‘gap-overlap’ pattern (a single gap and overlap is known as the D-period) to form microfibrils, which aggregate to form fibrils, as shown in figure 1. The tropocollagen molecules twist at a higher level to form a left-handed quasi-helical structure, with most of the twist occurring in the gap region [1].

To reinforce the structure of collagenous tissue and achieve a mechanical competence that combines both high strength and fracture toughness, nanometre-sized particles of carbonated hydroxyapatite (HA) are incorporated into the collagen fibrils through the process of mineralization. Mineralization of collagen is a fundamental event occurring during the development, adaptation and ageing of mammals and many other living organisms [2,3]. Mineral deposition induces large contractile stresses in the collagen fibrils and results in a rapid increase in the stiffness of the entire mineralized collagen composite [2–7]. It is, therefore, critically important to understand the impact of mineralization on the collagen structure changes for tuning the mechanical properties of biomaterials (e.g. tissue-engineered constructs [8–10]) and addressing clinical challenges (e.g. tendon-bone enthesis repair [11,12]). However, despite significant efforts in investigating the alterations in collagen structure and cross-linking that take place during mineralization [13,14], the influence of mineralization on the molecular organization and heterogeneity of the protein remains unclear.

Synchrotron X-ray scattering has been used extensively in studies of mineralized tissues to elucidate the hierarchical structure of both mineral and collagen [2,4,5,7,15–17]. The technique relies on the Rayleigh scattering of high-brilliance X-rays by electrons in a specimen, which gives rise to shape and diffraction features that are collected by a detector. Analysis of these features allows the quantification of the crystal lattice and shape/size of mineral platelets as well as the quasi-crystalline packing of fibrils and molecules comprising the collagenous matrix. As synchrotron technology has developed, the available flux, beamline optics and motor control have improved, permitting increasingly ambitious experiments. In this study, we used a beamline equipped for high-throughput macromolecular crystallography to obtain high-resolution synchrotron X-ray diffraction (XRD) data from turkey leg tendon (TLT) specimens. Through comparison with X-ray radiographs, changes in the hierarchical structure of collagen were quantified as a function of the degree of mineralization in different regions of the TLT samples.

Second harmonic generation (SHG) is a coherent nonlinear optical process in which a high-intensity laser induces electrons confined in non-centrosymmetric molecular potentials to radiate light at twice the incident laser frequency [18]. The coherent nature of the SHG process is such that the total signal recorded from one illuminated volume of the sample derives from the coherent addition of all individual sources of SHG, known as harmonophores, within the volume. As a result, the SHG signal is fundamentally related to the structure and organization of the sample on length scales that are much smaller than the wavelength of light [18]. In polarization-resolved second harmonic generation (pSHG) microscopy, a series of SHG images is acquired from the same region of the sample. Between each acquisition, the orientation of the polarization axis of linearly polarized light used to illuminate the sample is rotated through an angle in the sample plane. The degree to which the SHG intensity of each pixel in the image series is modulated by the change in polarization angle is understood to be a measure of the sample organization at molecular length scales [19].

The fibrillar collagens are an efficient source of SHG signal and increasingly SHG microscopy is regarded as the gold-standard for imaging its microscopic structure [20]. In collagen, SHG is presumed to originate from the regularly spaced, non-centrosymmetric peptide bonds that lie along the tropocollagen backbone [21,22], although recent research has suggested that methylene groups also contribute to the collagen SHG emission [23,24]. pSHG has been used extensively to investigate the molecular organization of collagenous tissues [25–31]. However, in materials with a complex hierarchical structure such as fibrillar collagen, the interpretation of the length scale at which the material structure is probed by pSHG remains a challenging question. The reason for this uncertainty is that the pSHG signal is determined by several factors, including the organization of the SHG harmonophores within each tropocollagen molecule as well as the organization of tropocollagen molecules within microfibrils and the organization of microfibrils within fibrils [32]. Despite the first demonstration of pSHG in collagen occurring over 40 years ago [33], there remains a paucity of experimental evidence that addresses the length scale in the hierarchical structure of collagen probed by pSHG [34]. In the work reported here, we use the mineralization-driven changes in TLT collagen organization at a molecular level to qualitatively assess the sensitivity of pSHG-derived parameters to these molecular level changes.

In this study, we examined the organization of type I collagen at molecular and fibrillar scales in normally mineralizing TLT. TLT has been widely used as a model system for studying mechanisms of collagen mineralization in vertebrate tissues [5,7,15,35–37], partly because it comprises collagen fibrils arranged in predominantly parallel arrays rather than in more complex twisted assemblages found in bone and other biomineralizing systems [35]. Mineralization begins in the distal aspect of the leg tendon and continues proximally from that origin, leading to a defined spatial gradient in mineralization that can be followed and analysed along the tendon length. Therefore, the early (newer mineral deposition)-to-late (older mineral deposition) sites of mineralization occur respectively along proximal-to-distal regions of the tendon. We obtained high-resolution synchrotron XRD data from zones with different degrees of mineralization along the lengths of normally mineralizing TLT at significantly higher resolution than that achievable on a standard small-angle X-ray scattering (SAXS) beamline. The position and angular dependence of features associated with molecular and fibrillar structure of type I collagen were quantified to determine trends in their size, orientation and order. Furthermore, we used the same regions of the TLT samples to investigate the impact of mineralization on pSHG measurements of collagen organization. Upon comparing data from tendon regions that represent different stages of mineralization, both biophysical imaging methods revealed a positive correlation between the extent of mineralization and the organization of collagen molecules.

## 2. Methods

### 2.1. Specimen preparation

**Figure 2.**
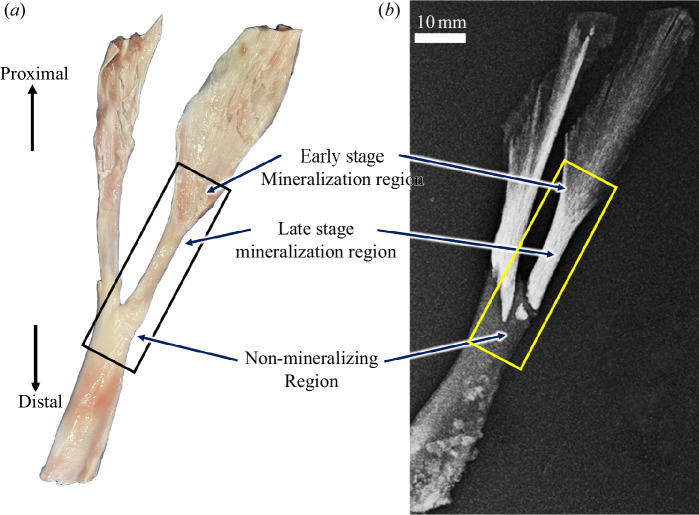
A light photomicrograph (*a*) and an X-ray radiograph (*b*) of a portion of the gastrocnemius tendon from the leg of a domestic turkey. Mineralization of the tissue begins near the point of bifurcation of the gastrocnemius into two segments and progresses in a distal-to-proximal direction along the lengths of both segments. Each segment mineralizes independently. The region of the tendon distal to its point of bifurcation does not mineralize (NM region). The regions of each segment near the point of bifurcation represent late stage mineralization, and regions more proximal along the longitudinal plane of each segment represent early stage mineralization [36].

Fresh legs from five domestic turkeys, 18–20 weeks old, were obtained from local meat suppliers. The gender of the turkeys could not be determined. The gastrocnemius tendons, which present a continuous and complete spatial and temporal pattern of mineralization of the tissue along their length, including regions that never mineralize and those that represent early, intermediate and late stages of mineralization [35], were carefully dissected in phosphate-buffered saline (PBS) to maintain a hydrated state (figure 2*a*). The tendon specimens were first imaged using X-ray radiography to qualitatively assess the spatial distribution of mineralization (figure 2*b*). Non-mineralizing (NM) tendon regions and regions of early mineralization (EM) and late (older) mineralization (LM) were identified in each sample using the normalized greyscale value in the radiographs. These three regions of the tendons from four of the five legs were subsequently trimmed into 40 mm × 10 mm × 1.5 mm segments. The dissected samples were used for pSHG scanning. One tendon was kept intact for synchrotron XRD. All dissected tendon segments were placed in holders and covered with optimal cutting temperature (OCT) compound (CellPath Ltd, Newton, Wales, UK), then frozen in a cryostat (OTF5000, Bright Instruments, Huntingdon, UK) at −40°C over ~4–5 min and stored at −20°C prior to experimentation. The intact tendon was immersed in OCT and frozen and stored in the same manner.

### 2.2. Synchrotron X-ray diffraction

**Figure 3.**
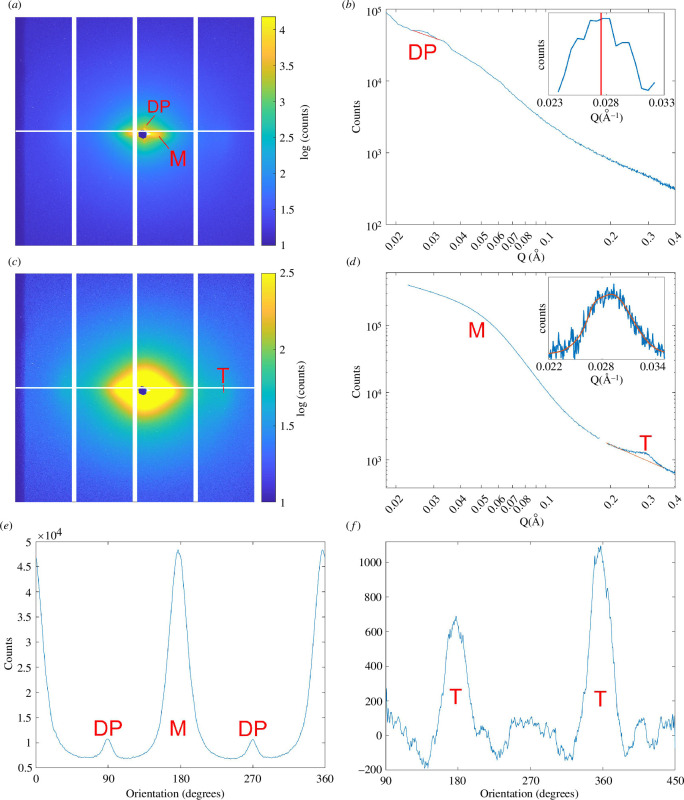
XRD analysis. (*a*) An example of an XRD scatter pattern with a threshold set so that the collagen D-period and mineral features (DP and M, respectively) are visible. (*b*) Radial intensity plot about the preferred fibril orientation, with the D-period peak (DP) highlighted and an inset showing the background-subtracted peak and its mean value. Note that the peak of the intensity plot is blunter than normal because of the divergent beam used for analysis. (*c*) The same scatter pattern as in panel A but with a threshold set so that the tropocollagen intermolecular feature (T) is visible. (*d*) Radial intensity plot orthogonal to the preferred fibril orientation with the mineral (M) and tropocollagen (T) peaks highlighted and an inset showing the background-subtracted peak and Gaussian fit. (*e*) Azimuthal distribution at a *Q*-value of 0.27 nm^−1^ (real spacing of 22.4 nm), showing peaks associated with mineral and D-spacing. (*f*) Azimuthal distribution at a *Q*-value of 2.7 nm^−1^ (real spacing of ~2.3 nm), showing peaks associated with tropocollagen intermolecular spacing.

XRD measurements from a single tendon specimen were acquired using the VMXi beamline at Diamond Light Source (DLS, Didcot, UK). The sample was brought to DLS in an insulated container and thawed at room temperature for ~1 h. The tendon was subsequently immersed in PBS for an additional 1–2 h, then removed from PBS and suspended in the XRD chamber for scanning over a total period of ~30 min. X-ray scatter patterns were acquired using an Eiger2 4M detector (Dectris, Baden, Switzerland) across the intact tendon in a snake scan with horizontal (*x*) increments of 20 µm and vertical (*y*) increments of 100 µm. The exposure time per scatter pattern was 2 ms, with the total projection comprising 522 500 scatter patterns. The beamline was configured to a photon energy of 12 658 eV, or a wavelength of 0.979 Å, and a divergent beam profile (divergence angle 1.375 mrad, focused using Kirkpatrick–Baez mirrors) with a spot size of approximately 10 µm × 10 µm at the sample. The detector was positioned 700 mm from the sample, providing a usable wavevector (*Q*) range of 0.2–5 nm^−1^ over a 2068 × 2162 pixel image (example images shown in figure 3*a,e*). Beamline flux was carefully monitored and then calibrated such that repeat images over the same tissue region produced patterns with no discernible differences. This approach mitigated the possibility that longer XRD scan times might dry and gelatinize the tendon collagen. Additional details about the VMXi beamline can be found in Sanchez-Weatherby *et al*. [38]. This setup allowed the visualization of diffraction peaks associated with the D-period of the tendon collagen fibrils and the intermolecular spacing of collagen molecules, hence providing information at fibrillar and molecular length scales simultaneously.

X-ray scattering analysis was carried out using a combination of DAWN (Data Analysis WorkbeNch, Didcot, UK) and MATLAB (version R2022a, MathWorks Inc., Natick, MA, USA). The detailed descriptions of data reduction and structural analysis have been published previously [38,39]. To improve signal:noise ratio, data were binned horizontally to obtain a dataset comprising 104 500 scatter patterns spaced 100 µm apart in *x* and *y*. Intensity (*I*) versus wavevector (*Q*) plots (figure 3*b,d*) were generated by converting the images into polar coordinates and integrating over the azimuth. The *I*–*Q* plots allowed quantification of the *Q* ranges of the third meridional peak of the collagen D-period (0.24–0.32 nm^−1^, corresponding to a real-space size of ~22.4 nm) and the intermolecular peaks (1.9–3.5 nm^−1^, corresponding to a real-space size of ~2.3 nm). Background subtraction (assuming a Porod-shaped background of the form $I∼Q^{-4}$ ) was handled by fitting a linear polynomial to a plot of $\ln\left(I\right)$ versus $\ln\left(Q\right)$ to data on both sides of the peak, subtracting the polynomial and raising the background-subtracted data to an exponential (see figure 3*b,d* insets).

For the intermolecular spacing, the resulting peak was fitted with a Gaussian function to find the peak value. The D-period peak, which is usually a sharply defined feature occupying a narrow *Q*-range on highly collimated SAXS beamlines, was broadened by the divergent beam and more variable in shape, presumably because of its close proximity to the beam stop and associated flare. The mean value of the peak was therefore used as a measure of the D-period (as opposed to the statistical mode, which is the typical method in SAXS analysis). The angular dependency of the two features was quantified by splitting the image into 720 0.5° azimuthal segments and repeating the background subtraction for each segment. The total peak signal was found for each segment and used to produce plots of the polar dispersion of each feature (figure 3*e,f* ). The broad, low-Q feature associated with mineral scatter overlapped the meridional D-period diffraction peak in Q, but it covered an azimuthal range perpendicular to the predominant fibril orientation and so did not significantly affect the analysis. For each scatter pattern, the radial position of the third meridional diffraction peak provided a measure of the D-period and the full width at half maximum of its azimuthal distribution was used to characterize the organization and orientation of fibrils.

The intermolecular spacing was quantified by finding the radial position of the corresponding diffraction peak, and its azimuthal distribution was used to characterize the orientation and organization of tropocollagen molecules. Estimation of supramolecular twist angle was carried out through comparison of fibril and molecule orientation dispersion data, using a scattering model that has been described elsewhere [39]. Because of the close proximity of fibril features to the beam stop and the divergent beam, specific values of the collagen fibril twist angle could not be reliably obtained, but the data were of sufficient quality to provide a measure of the relative trends between tendon regions.

### 2.3. Polarization-resolved second harmonic generation microscopy

Four tendon samples were imaged using pSHG microscopy. Each sample was thawed at room temperature, placed on a microscope slide, submerged in PBS and mounted underneath a cover-slip prior to being scanned for 4–5 min. A single sample was rescanned 2 h later when the specimen was air-dried. Observed changes in the respective scanned and rescanned data were marginal (electronic supplementary material, S2, figure 1). pSHG was performed using a custom-built laser scanning microscope. A mode-locked Ti:sapphire laser (MIRA 900, Coherent, Santa Clara, CA, USA) emitting 150 fs pulses at a central wavelength of 810 nm provided the illumination for these experiments. Prior to entering the scanning unit of the microscope, the polarization of the laser beam was adjusted using a pair of achromatic waveplates, each mounted in a dedicated electronic rotation stage (ELL14, Thorlabs, Newton, NJ, USA). Following a procedure [21] to change the angle of polarization in the sample plane of the microscope and simultaneously to compensate for unwanted birefringence of the microscope apparatus, the orientation of the half waveplate (AHWP05M-980, Thorlabs) and quarter waveplate (AQWP05M-980, Thorlabs) were adjusted in concert according to a look-up table. The look-up table was calibrated by measuring the optical polarization state at the back focal plane of the microscope objective lens using a rotating linear polarizer (LPNIRB050-MP2, Thorlabs) and a power meter (S170C, Thorlabs). By coordinating the orientation of these two waveplates, the polarization was maintained at >90% linearly polarized for all measurements [40].

Laser light was focused onto the sample using a long working distance water immersion lens (NA = 1.05, XLPLN25XWMP, Olympus, Tokyo, Japan), with an average power of 30 mW. The sample emission was collected in the epi-direction and spectrally separated from the laser light using a long-pass dichroic beam splitter (670DCXR, Chroma Technologies, Bellows Falls, VT, USA). A further dichroic beam splitter (Di02-R405, Semrock, IDEX Corporation, Lake Forest, IL, USA) was used to resolve the sample emission into two spectral channels, one for SHG and the other for two photon-excited fluorescence (TPEF). The TPEF channel was not used in the current study. Collagen SHG emission was detected using a photomultiplier tube (R3896, Hamamatsu Photonics, Shizuoka, Japan) coupled with a narrow band optical filter (FF01-405/10, Semrock, IDEX Corporation).

To probe the polarization response of the SHG emission, a series of SHG intensity images was recorded using different angles of linear polarization. Each 512 × 512 pixel image encompassed a field of view of 384.5 µm × 384.5 µm. Following the acquisition of an image, the half and quarter waveplates were used to rotate the polarization through an angle of 15° in the sample plane before the acquisition of the next image was triggered. A complete pSHG acquisition consisted of 12 SHG images, stacked to form an array of 512 × 512 × 12 intensity values. For each TLT sample, pSHG acquisitions were acquired from six locations in the NM region and four different locations in the EM and LM regions. An intensity threshold was applied to all SHG images to mask out regions with insufficient SHG intensity to produce reliable results from the subsequent pSHG analysis.

The pSHG stacks were analysed using a generic model that avoids the need to invoke a particular distribution of collagen SHG harmonophores *a priori* [19]. Following this approach, the intensity $I\left(\alpha \right)$ as a function of polarization angle (*α*) at each pixel was projected onto a sum of circular functions:


(3.1) $$I\left(\alpha \right)=a_{0}+I_{2}\cos\left(2\left(\alpha -\varphi_{2}\right)\right)+I_{4}\cos\left(4\left(\alpha -\varphi_{4}\right)\right)$$

**Figure 4.**
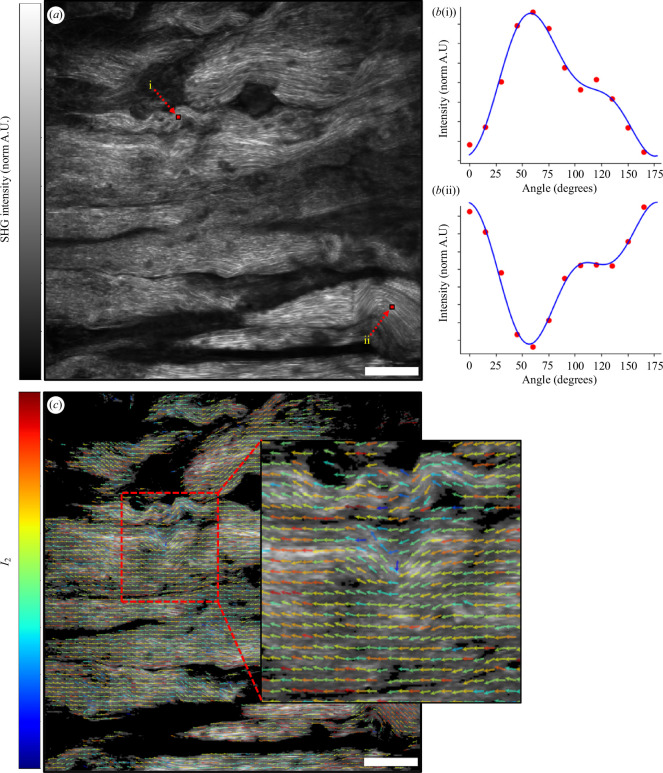
Overview of pSHG microscopy. (*a*) A single SHG image of type I collagen fibres in the NM region of a TLT. (*b*(i) and *b*(ii)) Plots of the normalized SHG intensity as a function of polarization angle for the pair of pixels highlighted in *a*. The normalized intensity measurements (red markers) were fitted using an analytical function (blue line) consisting of a sum of circular functions (see equation (3.1)). (*c*) The SHG image from *a*, overlaid with coloured arrows. The direction of each coloured arrow indicates the dominant direction around which the SHG harmonophores were aligned ${(\varphi }_{2})$ , and the colour of each arrow depicts the degree of organization ${(I}_{2})$ . For clarity, arrows are only printed for every 10th pixel. Scale bar = 50 µm.

Here, $a_{0}$ is the mean value of $I\left(\alpha \right)$ , $\varphi_{2}$ denotes the dominant orientation of the distribution of SHG harmonophores and $I_{2}$ is a measure of the degree of organization around this dominant direction. It should be noted that larger $I_{2}$ values correspond to higher degrees of organization in the sample [27,28,41,42]. The parameters $I_{4}$ and $\varphi_{4}$ are the organization and orientation signatures, respectively, of the more complex fourth-order dependence of SHG intensity on polarization angle and were not used in this study. This analysis produced 512 × 512 pixel maps of $I_{2}$ and $\varphi_{2}$ that illustrated the spatial distribution of collagen fibril organization and direction in the sample (see figure 4*c*). The mean of $I_{2}$ was calculated for each pSHG acquisition and these means were used to calculate the overall mean and standard deviation of $I_{2}$ for the different degrees of mineralization present in a sample.

### 2.4. Statistical analysis

To quantify significance in measurements across the three TLT regions of interest, a one-way ANOVA with Tukey–Kramer post hoc test was performed. For the XRD analysis, we evaluated several variables, namely, the collagen D-period, intermolecular spacing, fibril dispersion, molecular dispersion and supramolecular twist, by extracting these values from individual pixels. The values were then compared to ascertain significant differences among the three ROIs. In the case of pSHG, the dataset consisted of mean $I_{2}$ values, which were generated from a total of 56 scans across four samples. For each sample, as also noted above, six distinct scans were obtained over various locations within the NM tendon region and four scans were obtained over different locations for both the LM and EM regions. All statistical analyses were carried out using Matlab.

## 3. Results

### 3.1. XRD data

**Figure 5.**
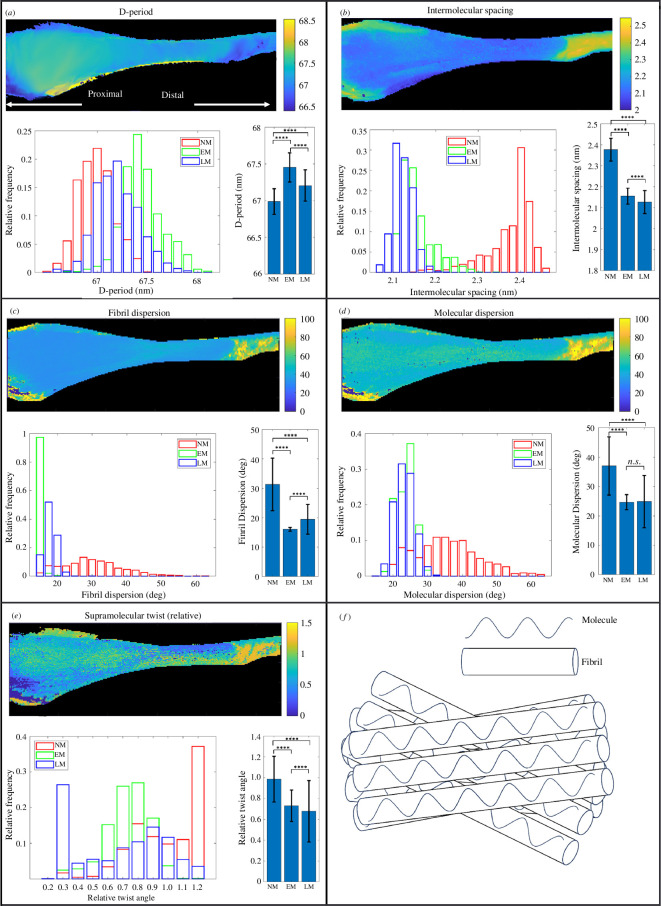
(*a–e*) XRD metrics shown spatially in surface plots, with histograms depicting the statistical distribution associated with extent of mineralization and bar charts showing the mean and standard deviation. Metrics displayed are (*a*) the collagen D-period, (*b*) collagen intermolecular spacing, (*c*) fibril dispersion, (*d*) molecular dispersion and (*e*) relative supramolecular twist. The proximal and distal directions along the tendon are indicated in panel *a*. (*f*) Diagram illustrating how the molecular dispersion is a function of both the intrafibrillar molecular orientation distribution and the dispersion of fibrils. The values exhibit statistical differences, indicated by ****p < 0.0001, and no significant statistical difference, indicated by n.s. For each region, the number of data points varies: 1071 in the LM region, 1372 in EM and 1059 in NM. Consequently, a one-way ANOVA was performed followed by a Tukey–Kramer post hoc test. Statistically significant differences were identified between NM and EM, EM and LM, and NM and LM, across the variables of the D-period, fibril dispersion, molecular dispersion and supramolecular twist. For molecular dispersion, statistically significant variations were observed between NM and EM as well as between NM and LM; however, no statistically significant difference was found between EM and LM.

The spatial distribution of the type I collagen D-period, intermolecular spacing and their associated azimuthal dispersions are illustrated in figure 5*a*–*d*. Beneath their respective heat maps, histograms of XRD structure metrics for each of the three tendon regions, NM, LM and EM, are depicted. Using one-way ANOVA followed by a Tukey–Kramer post hoc test, statistically significant differences (*p* < 0.0001) were found among NM, EM and LM for the variables of the collagen D-period (figure 5*a*), intermolecular spacing (figure 5*b*), fibril dispersion (figure 5*c*) and supramolecular twist (figure 5*e*). For molecular dispersion (figure 5*d*), statistically significant differences (*p* < 0.0001) were observed between NM and LM as well as between NM and EM, with no significant difference between EM and LM. These results underscored the need for a more thorough investigation of the mean differences in these XRD quantities across the three tendon regions. The D-period (figure 5*a*) did not exhibit a continuous trend with mineralization, with NM presenting the lowest mean value (66.92 ± 0.19 nm), followed by LM (67.21 ± 0.23 nm) and then EM (67.46 ± 0.22 nm) (*p* < 0.0001). The intermolecular spacing (figure 5*b*) in NM (2.37 ± 0.05 nm) was significantly greater (*p* < 0.0001) compared to EM (2.16 ± 0.03 nm) and LM (2.13 ± 0.05 nm), as well as EM compared to LM. Similarly, the measurements of azimuthal dispersion for fibrils (31.19° ± 9.71°) and molecules (37.43° ± 9.07°) in NM (figure 5*c,d*, respectively) were significantly greater (*p* < 0.0001) compared to those in EM (15.31° ± 0.90° for fibrils and 24.55° ± 2.95° for molecules) and LM (19.88° ± 4.67° for fibrils and 24.96° ± 9.54° for molecules). However, the mean values showed relatively small differences between EM and LM. Regarding the dispersion of molecules, the mean difference between EM and LM was marginal. It should be noted that the azimuthal dispersion of molecules is a function of both the intrafibrillar molecular structure and the orientation dispersion of the fibrils within the imaged volume (illustrated in figure 5*f*), which for a 10 µm × 10 µm beam passing through a sample ~1 mm thick is likely to include contributions from many thousands of fibrils. To isolate the intrafibrillar molecular structure, we calculated the mean supramolecular twist and normalized it relative to the NM region. The model found that the relative twist angle (figure 5*e*) was significantly greater (*p* < 0.0001) in the NM (0.98 ± 0.23) region than in the EM (0.72 ± 0.16) and LM (0.68 ± 0.28) regions, with a smaller change (0.04) from EM to LM (*p* < 0.0001) (figure 5*e*).

### 3.2. pSHG data

**Figure 6.**
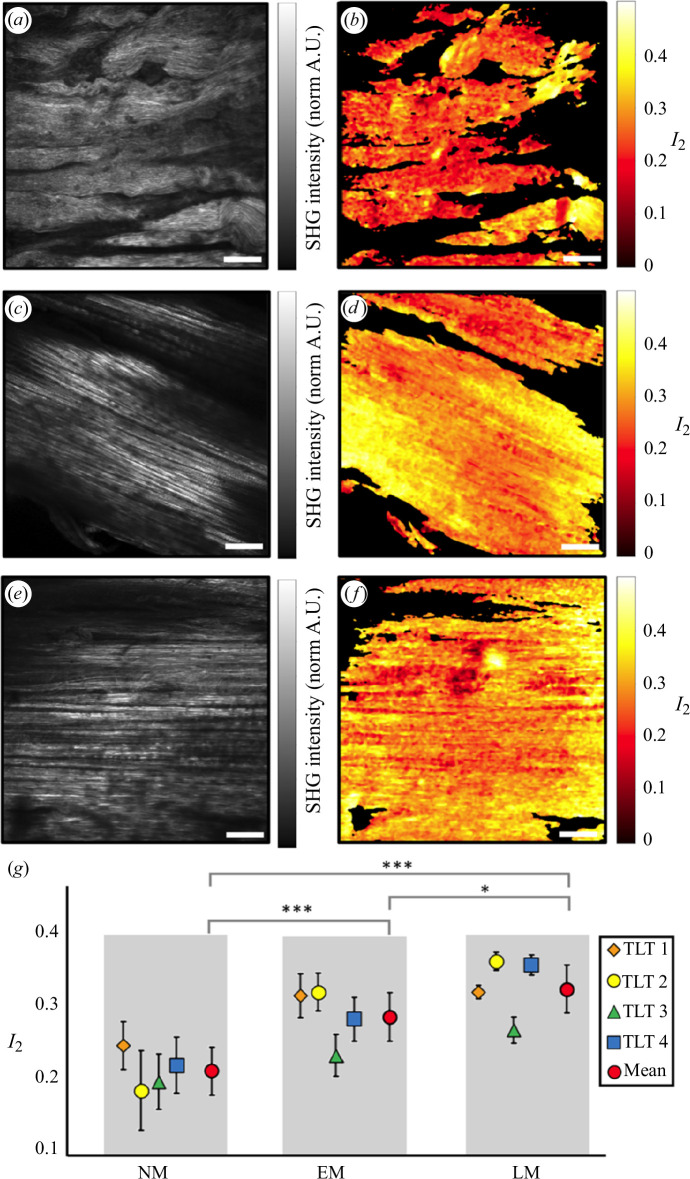
(*a*, *c*, *e*) SHG intensity images acquired from the NM, EM and LM regions, respectively, of a TLT. (*b*, *d*, *f*). Spatial distributions of the pSHG organization parameter $I_{2}$ for the fields of view depicted in *a*, *c* and *e*, respectively. (*g*) Intrasample variability of the mean and standard deviation of $I_{2}$ in the NM, EM and LM regions. The red markers and their error bars indicate the mean and standard deviation of $I_{2}$ measured in each region across all samples. The mean values show significant differences (*p < 0.05, **p < 0.01, ***p < 0.001). Scale bars = 50 µm.

Example SHG intensity images and their corresponding $I_{2}$ maps acquired from the NM, EM and LM regions of a TLT sample are shown in figure 6*a,b,c,d,e,f*. Figure 6g presents the summary results of pSHG analysis of the three different regions (NM, EM and LM) from all tendon segments (TLT1–4) investigated in this study. Plots of the mean $I_{2}$ values for the regions and segments revealed several interesting aspects of the data. First, there were differences in individual measured $I_{2}$ values within each of the three regions as would be expected. These data led to mean $I_{2}$ values determined to be 0.21 ± 0.044 for NM, 0.29 ± 0.043 for EM and 0.33 ± 0.038 for LM (figure 6*c*). Second, from one-way ANOVA, statistically significant differences (*p* < 0.05) were found among NM, EM and LM for these mean $I_{2}$ values. The subsequent Tukey–Kramer post hoc analyses indicated statistically significant differences between NM and EM (*p* < 0.001), EM and LM (*p* < 0.05) and NM and LM (*p* < 0.001). Finally, these statistically significant differences represented a consistent trend of increasing $I_{2}$ as a function of increasing mineralization (NM to EM to LM) for all the tendon samples.

## 4. Discussion

In this study, synchrotron XRD and pSHG have been applied to quantify changes in the hierarchical structure of fibrillar collagen associated with mineralization. TLT was used as a model system, and measurements with the two techniques above were acquired in tendon regions characterized by the absence of mineral altogether as well as by early and late stages of mineral formation. The most noteworthy changes appear to be at the molecular scale of structural hierarchy, where the presence of increasing mineralization coincides with an increased $I_{2}$ and decreased supramolecular twist, a result for both parameters suggesting an increase in structural order.

It is widely recognized that early stages of mineral deposition in collagen occur in part in the intrafibrillar gap region [43–45] of the protein and, as mineralization progresses, the mineral content, predominantly HA, increases both along and perpendicular to the fibril axis. Such mineral deposition can lead to a broad range of collagen–mineral structures, which can be categorized in terms of axial and lateral mineral packing [2,35,37]. Axial packing involves a staggered molecular and higher ordered arrangement of mineral, accentuating the characteristic collagen D-period already present as a consequence of molecular, microfibrillar and fibrillar self-assembly of the protein [46,47]. Lateral packing, on the other hand, pertains to the side-by-side arrangement of collagen molecules, between which mineral particles grow perpendicularly to the longitudinal axis.

Differences in the collagen intermolecular spacing and D-period between regions offer insights into collagen–mineral interactions. The intermolecular spacing of collagen is sensitive to hydration [48]. As with the decrease in collagen intermolecular spacing with increasing mineralization, the loss of intra- and interfibrillar water with increasing mineral presence in the tendon tissue may provide an explanation for the observed changes in dispersion [2]. Besides hydration [48] the D-period is also sensitive to fibrillar strain [39], supramolecular structure [39] and mineralization [49–51]. A recent study examining microstructural changes during tendon mineralization [50] observed that the mineralized region of the tissue exhibited a decrease in D-spacing relative to its unmineralized region. Earlier, Xu *et al*. [51]**,** investigating chemical aspects of the interaction between mineral and the intermolecular structure of collagen, reported that the gap regions elongated because of the presence of mineral particles. This result is consistent with the findings of the current study.

In this context, one may speculate as to the nature of the observed collagen–mineral changes in TLT. In an initial sense, the increase in collagen D-period with the onset and progression of mineralization found in this study would support the hypothesis that mineral deposition in the gap regions may push collagen molecules apart, expanding their axial separation while collagen bonds and crosslinks are maintained. However, the result that regions of LM present a smaller D-period than that measured in regions of EM suggests a more complex collagen–mineral relationship. In this instance, a possible explanation could involve the concept that increasing mineral growth and development during late-stage mineralization disrupts bonds and cross-linking within collagen, causing a collapse in axial separation of the molecules and a decrease in D-period. Envelopment or occlusion of collagen peptide chains and molecules by increasing mineral may also compress and reduce the axial separation of the D-period. Compounding these conjectural possibilities, the D-period is likely also affected by changes in intrafibrillar water, which presumably would be expressed or lost as mineral nucleation, growth and development progressed within collagen [52,53]. In this circumstance, the D-period might be expected to decrease as mineral mass increases. Whatever the true response may be of the collagen D-period to mineral presence in the tissue, further application of synchrotron XRD offers opportunities for greater understanding of collagen–mineral interactions at finer levels of structural hierarchy.

Supramolecular twist refers to the distinct arrangement in the molecular packing topology of the collagen molecule. In this structure, adjacent molecules organize to form a super-twisted right-handed microfibril [1]. This occurrence takes place at a dimension larger than that of the tropocollagen triple helix, yet smaller than the fibrillar crimp. The estimation of the supramolecular twist was achieved through a comparison of fibrillar and molecular dispersions [54]. It was found to decrease significantly with mineralization, implying that the presence of mineral straightens or elongates the supramolecular structure. The scattering model employed in this study assumes a helical twist of supramolecular structures for mathematical simplicity, but it has been postulated [1] that the majority of twist/deflection at the supramolecular scale occurs in the gap region, which is where mineral is initially deposited. Furthermore, it has been suggested that mineral ions may preferentially bind to more tortuous regions on the collagen molecule that are devoid of proline and hydroxyproline [55], effectively lengthening the axial rise per residue and thereby stretching the D-period. Quantification of supramolecular twist also provides a measure of intrafibrillar structure for comparison with pSHG measurements (in which each voxel was approximately 0.75 µm × 0.75 µm × ~2 µm and therefore likely to sample only a small number of mostly parallel collagen fibrils).

Although SHG has been widely used in many fields for visualizing fibrous collagen networks [56–58], this work represents the first time SHG microscopy has been used to study the impact of mineralization on collagen organization in TLT. A significant intersample variability in mean $I_{2}$ values was measured from tendon regions with a similar degree of mineralization (NM and EM and LM). This result would be expected as the turkeys from which tendons were obtained are each distinct in growth rates and maturation, and likely in age and gender as well, all features reflected in the properties, including *I*_2_, of their tissues. Despite such variability, significant differences in $I_{2}$ as a function of collagen mineralization were observed, a result suggesting that pSHG is compatible with probing mineralization-induced changes in collagen organization.

An important aspect of this study was to provide experimental insight into the length scale at which pSHG probes collagen organization. We compared qualitatively the pSHG and XRD measurements, the latter of which is regarded as a gold-standard method for determining molecular level changes in collagenous tissues. The trend of increasing $I_{2}$ as a function of mineralization using pSHG complements the trend of decreasing relative supramolecular twist angle as a function of mineralization found using XRD. This approach in turn provides an experimental basis for the long-held assumption that pSHG is sensitive to the molecular organization of collagen. It is important to note that, while pSHG is a depth-resolved technique that acquires data from a single plane within a sample, XRD measurements are an integral of the full sample depth. A more quantitative comparison would be provided by acquiring data with the two imaging modalities from the same samples and by increasing the statistical power of the study. From a physicochemical and biochemical standpoint, these summary results demonstrate a complex interplay between several aspects of collagen molecules and the onset and development of mineral deposition. The data are intriguing and provide insights into collagen–mineral interactions revealed uniquely by synchrotron XRD and pSHG. These methods will be applied in future studies to gain even more understanding of the behaviour of collagen as it becomes progressively mineralized in model tissues *in vivo*.

While the present study yields valuable insights into the effects of mineralization on collagen structural organization, it is worth noting several factors that may influence and limit the broader applicability and interpretation of the results presented here. First, there is a question as to whether the NM regions of a TLT distal to the point of bifurcation of the gastrocnemius tendon (NM in this study) and NM regions even more proximal to EM maintain collagen with similar or the same microstructural properties. It is important to examine the latter because these very proximal regions will eventually mineralize as the turkey develops and these regions may be distinct from the collagen comprising NM. While no direct evidence exists to suggest significant microstructural differences between these two areas, Chen *et al*. [59] have reported differing gene expression patterns between regions represented by NM, LM and EM. In this instance, additional pSHG scans were performed on one tendon sample to compare the $I_{2}$ values in its NM and in its very proximal NM regions. Detailed results (see electronic supplementary material, file S1) indicate no significant variation in $I_{2}$ values between the NM regions. Second, scanning conditions differ between XRD and pSHG acquisitions and in particular XRD requiring longer scan times which may ignificant change in the sample hydration state. To assess the effect of sample drying on collagen organization, pSHG scans were carried out before and after a 2 h interval. A minor shift in the mean $I_{2}$ value (0.016) was observed however, the change was smaller than the observed variation (0.11) between highly mineralized and NM regions. Details can be found in electronic supplementary material, file S2. Third, intersample XRD measurements have potential benefits in providing more comprehensive understanding and greater insight related to the topic areas of this work. However, the limited availability of beamline time restricted analysis to a single one sample in the present study. Nevertheless, the results obtained here provide crucial preliminary data, serving as a foundation for future studies that will use a broader range of samples.

It should also be mentioned that the XRD data, which were collected on a high-throughput crystallography beamline for high-resolution mapping, are subject to larger systematic errors than those expected from a SAXS beamline. Systematic errors associated with beam stop flare and the divergent beam suggest that absolute values for the D-period may differ slightly from the published literature. In this instance, the focus in this study was directed towards overall trends and relative changes. Finally, it should be noted that the tendon samples were frozen and stored for one to two weeks and then thawed before any analysis. Freezing was done in a cryostat using OCT compound to help mitigate potential freezing and ice crystal effects. While freezing and thawing of tendon samples may induce changes in collagen structure, XRD measurements of the D-period of the mineralized collagen in this study yielded 67 nm which has been observed in fresh tendon specimens by other XRD studies of TLT [15,35,60–62].

## 5. Conclusion

In this study, both pSHG and XRD were used to explore the impact of mineralization on the hierarchical organization of collagen. Measurements acquired using both methods showed that mineral content generally increases the structural order of collagen in a TLT model, but also hinted at a complex and multifaceted process of mineralization. A further noteworthy aspect of this investigation is the utility of pSHG as a versatile tool for imaging connective tissues. This fast, label-free and non-destructive imaging technique is particularly well suited for hierarchical studies that span the molecular and fibrillar scales of collagen. pSHG stands as a promising analytical tool to enable future research into the hierarchical structural changes in collagen resulting from not only mineralization, but also biomechanical phenomena, connective tissue pathology and associated therapies.

## Data Availability

pSHG and XRD raw data are available from Zenodo: https://zenodo.org/records/10979115 [63]. Supplementary material is available online [64].
